# Hypoxia-Inducible Factor in Renal Cell Carcinoma: From Molecular Insights to Targeted Therapies

**DOI:** 10.3390/genes16010006

**Published:** 2024-12-24

**Authors:** Giandomenico Roviello, Irene De Gennaro, Ismaela Vascotto, Giulia Venturi, Alberto D’Angelo, Costanza Winchler, Adriana Guarino, Salvatore Cacioppo, Mikol Modesti, Marinella Micol Mela, Edoardo Francini, Laura Doni, Virginia Rossi, Elisabetta Gambale, Roberta Giorgione, Lorenzo Antonuzzo, Gabriella Nesi, Martina Catalano

**Affiliations:** 1 Department of Health Science, University of Florence, 50134 Florence, Italy; martina.catalano@unifi.it; 2 Department of Experimental and Clinical Medicine, University of Florence, 50134 Florence, Italy; irene.degennaroaquino@unifi.it (I.D.G.); ismaela.vascotto@unifi.it (I.V.); giulia.venturi@unifi.it (G.V.); costanza.winchler@unifi.it (C.W.); adriana.guarino@unifi.it (A.G.); salvatore.cacioppo@unifi.it (S.C.); micol.modesti@unifi.it (M.M.); edoardo.francini@unifi.it (E.F.); roberta.giorgione@unifi.it (R.G.); lorenzo.antonuzzo@unifi.it (L.A.); 3 Department of Medicine, Sheffield Teaching Hospital NHS Foundation Trust, Sheffield S10 2JF, UK; alberto.dangelo@nhs.net; 4 Clinical Oncology Unit, Careggi University Hospital, 50234 Florence, Italy; melam@aou-careggi.toscana.it (M.M.M.); donil@aou-careggi.toscana.it (L.D.); rossiv@aou-careggi.toscana.it (V.R.); gambalee@aou-careggi.toscana.it (E.G.); 5 Department of Health Sciences, Section of Anatomic Pathology, University of Florence, 50139 Florence, Italy; gabriella.nesi@unifi.it

**Keywords:** renal cell carcinoma, belzutifan, HIF-2α targeting, VHL tumor

## Abstract

Mutations of the von Hippel–Lindau (*VHL*) tumor suppressor gene occur frequently in clear cell renal cell carcinoma (RCC), the predominant histology of kidney cancer, and have been associated with its pathogenesis and progression. Alterations of *VHL* lead to impaired degradation of hypoxia-inducible factor 1α (HIF1α) and HIF2α promoting neoangiogenesis, which is pivotal for cancer growth. As such, targeting the VHL-HIF axis holds relevant potential for therapeutic purposes. Belzutifan, an HIF-2α inhibitor, has been recently indicated for metastatic RCC and other antiangiogenic drugs directed against HIF-2α are currently under investigation. Further, clinical and preclinical studies of combination approaches for metastatic RCC including belzutifan with cyclin-dependent kinase 4–6 inhibitors, tyrosine kinase inhibitors, or immune checkpoint inhibitors achieved promising results or are ongoing. This review aims to summarize the existing evidence regarding the VHL/HIF pathway, and the approved and emerging treatment strategies that target this pivotal molecular axis and their mechanisms of resistance.

## 1. Introduction

Renal cell carcinoma (RCC) accounts for the 9th and 14th most prevalent cancer in men and women, respectively [1]. The most relevant RCC histology is clear cell renal carcinoma (ccRCC) accounting for approximately 80% of all cases [2]; however, other histologies were described such as papillary RCC and chromophobe RCC; lastly, only a small percentage presented as very rare histology such as transitional cell carcinoma, nephroblastoma or Wilms’ tumor, collecting duct tumors, renal sarcomas, and renal medullary carcinomas [2].

To date, it has been well known that the von Hippel–Lindau (*VHL*) tumor suppressor gene plays a major role in the development and progression of RCC [3]. The role of VHL is to act as an E3 ubiquitin ligase leading to the ubiquitination of the alpha subunit of hypoxia-inducible factor (HIF) in an oxygen-dependent fashion; this binding can regulate the angiogenesis because of normoxic conditions, where VHL degrades the α-subunit of HIF. Conversely, in hypoxic conditions, HIF is not degraded by VHL, and the HIF signaling cascade can promote the formation of blood vessels [3]. As VHL function is generally defective in RCC, this is a key oncogenic driver for RCC patients and explains the efficacy and activity of more antiangiogenetic drugs [4]. Based on these premises, over the years, several antiangiogenetic drugs have been approved for the treatment of RCC [4].

Another important target for RCC is related to immunotherapy; in fact, the efficacy and safety of the immune checkpoint inhibitor (ICI) anti-programmed death 1 (PD-1) nivolumab has been investigated in a large phase III trial [5]. Considering that antiangiogenic and immunotherapy both act on the tumor microenvironment and that immune-vascular cross-talk exists and has a synergistic immune effect [6], several phase III studies showed that the combination of antiangiogenic tyrosine kinase inhibitors (TKIs) and immunotherapies had shown increased efficacy than TKI monotherapy for the primary treatment of metastatic RCC (mRCC) [7,8,9].

In particular, pembrolizumab together with axitinib in the KEYNOTE-426; another combination, nivolumab and cabozantinib in the CheckMate 9ER study; and a further immunotherapy/TKI combination was evaluated in the CLEAR study (lenvatinib plus pembrolizumab). Moreover, the anti-cytotoxic T-lymphocyte antigen 4 (CTLA-4) ipilimumab combined with nivolumab was investigated in the phase III CheckMate214 trial [10]. Unfortunately, long-term responses are rare with a high rate of resistance, highlighting the need for novel strategies with the aim of improving the efficacy and survival of mRCC.

More recently, the role of the VHL-HIF pathway in RCC has been highlighted because of the possibility of targeting HIF activity with several drugs including, among these [11], belzutifan (MK-6482), a second-generation HIF-2α inhibitor that is already a therapeutic option for patients with metastatic RCC. This review will examine the role of the VHL-HIF pathway in RCC as well as the effects of anti-HIF-2α therapy.

## 2. The VHL/HIF Pathway: Structure, Function, and Pathophysiological Significance

The VHL/HIF pathway is a fundamental cellular mechanism for oxygen sensing and adaptation to hypoxic conditions. This pathway controls the expression of genes that are essential for cellular survival under low oxygen, influencing key processes like angiogenesis, erythropoiesis, and metabolic reprogramming. Dysregulation of the VHL/HIF pathway is implicated in various diseases, including several cancers and chronic kidney disease, where an abnormal oxygen response plays a pivotal role [12].

### 2.1. Structural Components of the VHL/HIF Pathway

The VHL/HIF pathway centers on the interaction between the VHL protein, a tumor suppressor, and HIF, a transcription factor complex crucial for hypoxia-responsive gene expression [13]. HIF itself consists of two primary subunits: an oxygen-sensitive HIF-α subunit and a constitutively expressed HIF-β subunit. Under normal conditions, HIF-α undergoes hydroxylation at specific proline residues by prolyl hydroxylase domain (PHD) enzymes [14]. This hydroxylation enables the VHL protein, as part of an E3 ubiquitin ligase complex, to recognize HIF-α, tagging it for proteasomal degradation [15]. Degradation prevents the accumulation of HIF-α, keeping the hypoxia-responsive genes inactive in oxygen-rich environments [16]. Prolyl hydroxylation of HIF-α requires molecular oxygen, iron (Fe^2+^), and 2-oxoglutarate as cofactors, making PHD enzymes key oxygen sensors within the VHL/HIF pathway [17]. Under normal conditions, continuous hydroxylation and degradation of HIF-α maintain the pathway in a repressed state, ensuring that hypoxia-induced genes are only expressed when genuinely needed [18].

### 2.2. HIF and Its Regulatory Mechanisms

**Figure 1:**
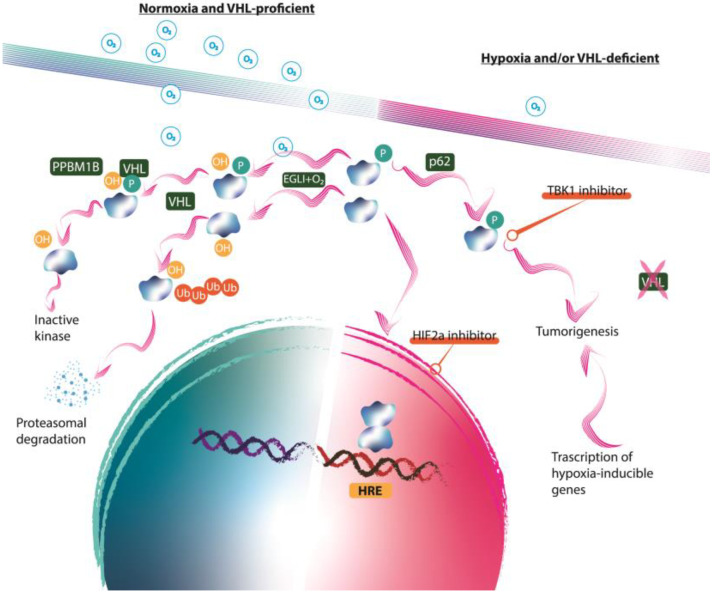
The role of HIFs in renal tumor development. The figure illustrates the involvement of HIFs in the pathogenesis of RCC. HIFs are key transcription factors activated under hypoxic conditions and are commonly dysregulated in RCC due to mutations in the VHL tumor suppressor gene. In normal oxygen levels, VHL targets HIF for proteasomal degradation. In RCC, the loss of VHL function leads to constitutive stabilization of the HIF-α subunit that accumulates and translocates to the nucleus and binds to HIF-β, forming an active transcriptional complex. This HIF complex binds to hypoxia-responsive elements (HREs) within the promoter regions of hypoxia-related genes, activating a broad transcriptional program that includes genes regulating angiogenesis, metabolism, erythropoiesis, and cell survival. Hypoxia-inducible factors (HIFs); renal cell carcinoma (RCC); von Hippel–Lindau (VHL).

In hypoxic conditions, reduced oxygen availability inhibits PHD activity, preventing HIF-α hydroxylation [19]. As a result, HIF-α is not recognized by VHL, thus escaping degradation. The stabilized HIF-α subunit accumulates and translocates to the nucleus, where it binds to HIF-β, forming an active transcriptional complex [20,21]. This HIF complex binds to hypoxia-responsive elements (HREs) within the promoter regions of hypoxia-related genes, activating a broad transcriptional program that includes genes regulating angiogenesis, metabolism, erythropoiesis, and cell survival [22] (Figure 1). Among these, it is worth mentioning the following genes:

- VEGF (vascular endothelial growth factor) promotes new blood vessel formation, enhancing oxygen delivery to hypoxic tissues.
- EPO (erythropoietin) stimulates red blood cell production, increasing the blood’s oxygen-carrying capacity.
- GLUT1 (glucose transporter 1) and other glycolytic enzymes shift cellular metabolism from oxidative phosphorylation to glycolysis, a process known as the “Warburg effect” in cancer, which allows cells to generate adenosine triphosphate (ATP) anaerobically.

These adaptations are critical for hypoxia but can drive disease progression when chronically activated in a dysregulated manner, as seen in certain cancers and fibrotic diseases [23].

### 2.3. HIF Isoforms and Their Functional Specificity

The main isoforms of HIF include HIF-1α, HIF-2α, and HIF-3α, each with specific regulatory roles. HIF-1α and HIF-2α are the most studied isoforms, with distinct but sometimes overlapping functions in response to hypoxia [24].

- HIF-1α is broadly expressed and is primarily responsible for regulating genes involved in glycolysis, facilitating anaerobic metabolism. It is crucial for acute responses to hypoxia and enables cells to meet energy demands when oxygen is limited [25].
- HIF-2α is more tissue-specific, primarily found in endothelial cells, interstitial fibroblasts, and certain types of cancer cells. It governs the expression of genes related to erythropoiesis (e.g., EPO), angiogenesis (e.g., VEGF), and iron metabolism, and is more prominent in chronic hypoxia adaptation. Notably, HIF-2α plays a crucial role in the progression of hypoxia-driven cancers like RCC [25].
- HIF-3α, though less well characterized, may act as a modulator rather than an activator of hypoxia responses, potentially inhibiting the function of HIF-1α and HIF-2α in specific contexts [26].

### 2.4. Pseudohypoxia: Pathological Activation of the VHL/HIF Pathway

Pseudohypoxia refers to the constitutive activation of HIF in normoxic conditions, a feature observed in certain cancers, such as ccRCC and pheochromocytomas/paragangliomas (PPGLs) [27]. In these cancers, mutations or deletions in the VHL gene result in a loss of function, preventing the degradation of HIF-α subunits under normoxia. This continuous HIF activation drives a pseudohypoxic response, promoting angiogenesis, cell proliferation, metabolic reprogramming, and anti-apoptotic mechanisms [22,27].

For instance, in ccRCC, the hypervascular nature of tumors is attributed to excessive angiogenesis driven by constitutively active HIF-2α [27]. This persistent activation not only supports tumor growth but also enables metastasis, making it a significant target for cancer treatments.

Research into the differential roles of HIF-1α and HIF-2α in tumor biology continues to drive the development of more selective therapies, as targeting HIF-2α alone may provide specific benefits without the broader metabolic effects associated with pan-HIF inhibition [28]. In addition to cancer, modulating the VHL/HIF pathway holds potential in treating conditions characterized by chronic hypoxia, such as pulmonary hypertension and chronic kidney disease.

## 3. Clinical Trials Involving Anti-HIF-2α

### 3.1. Belzutifan Monotherapy

**Figure 2:**
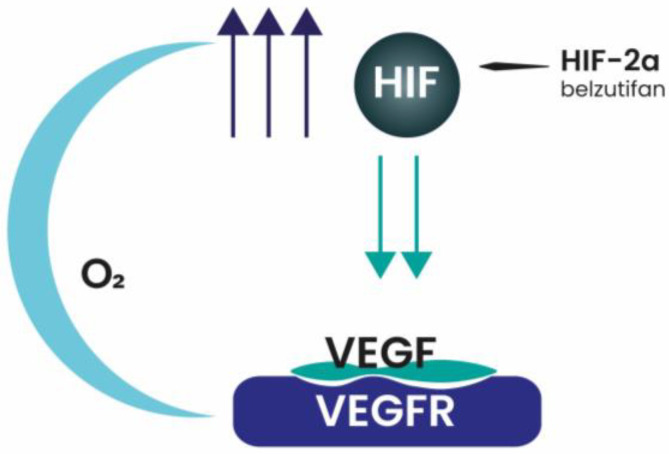
Belzutifan inhibits hypoxia-inducible factor-2 alpha (HIF-2α), a key transcription factor involved in cellular adaptation to hypoxia. By targeting HIF-2α, belzutifan disrupts tumor growth and survival pathways in hypoxia-driven cancers.

Belzutifan, a second-generation small-molecule HIF-2α inhibitor (Figure 2), has shown promise as monotherapy in treating advanced ccRCC across multiple clinical trials. In the phase I LITESPARK-018 trial (NCT04846920), patients who had experienced progression after previous systemic therapies were administered belzutifan in escalating doses to assess safety and pharmacokinetics.

The results highlighted a favorable safety profile, with a substantial proportion of patients experiencing manageable adverse events (AEs), such as anemia and fatigue, but without severe toxicity limiting further development. The primary outcome measure is the percentage of participants experiencing AEs, tracked over approximately 49.5 months. Secondary outcomes include pharmacokinetic assessments, such as the area under the plasma concentration–time curve (AUC) of belzutifan, measured at multiple intervals over the initial days of treatment.

In parallel, the outcomes of NCT04489771 further explored the anti-tumor efficacy of belzutifan, demonstrating notable clinical activity in heavily pre-treated patients, including partial responses and prolonged disease stabilization. Belzutifan was administered orally at 120 mg once daily. The study demonstrated a high overall response rate (ORR), with 49% of RCC tumors showing significant reduction in size, and a notable 63% response rate in hemangioblastomas. The median time to respond was around 3.1 months, showcasing its efficacy. Common adverse events (AEs) included anemia and fatigue, but these were manageable and consistent with the drug’s mechanism of action, targeting HIF-2α. The trial emphasized the drug’s potential to target the HIF pathway effectively, particularly in patients with VHL disease-associated ccRCC, where durable tumor responses were observed.

The phase II trial NCT04195750 reinforced these findings, underscoring a robust disease control rate (DCR) and durable responses, especially in patients who had limited options following standard treatments. This study specifically targeted patients whose disease had progressed despite prior treatment with both ICIs (such as PD-1/programmed death-ligand 1 (PD-L1) therapies) and VEGF tyrosine kinase inhibitors (VEGF-TKIs). Participants were randomly assigned to receive either 120 mg of belzutifan or 10 mg of everolimus daily. The trial’s primary objectives were progression-free survival (PFS) and overall survival (OS), with secondary measures including ORR, duration of response, safety profiles, and health-related quality of life (HRQoL) assessments. QoL outcomes were analyzed using validated instruments like the Functional Assessment of Cancer Therapy–Kidney Symptom Index—Disease-Related Symptoms (FKSI-DRS) and European Organisation for Research and Treatment of Cancer Core Quality of Life questionnaire (EORTC QLQ-C30) scales, which measure disease-related symptoms and overall physical functioning. The results showed that belzutifan significantly extended PFS compared to everolimus and presented a more favorable safety profile. Median OS was 21.4 months for belzutifan and 18.1 months for everolimus, although this difference did not reach statistical significance. AEs were common in both groups, with a slightly lower incidence of severe toxicities in the belzutifan arm. Notably, belzutifan-treated patients experienced fewer cases of hyperglycemia and stomatitis, but there was a higher occurrence of anemia compared to everolimus. These results support belzutifan as a promising treatment alternative for heavily pre-treated patients with advanced RCC, offering clinical benefits and improved tolerability compared to traditional options like everolimus.

These results collectively indicate that belzutifan, as a monotherapy, can provide significant clinical benefits, offering a new mechanism-based approach for ccRCC management that warrants further exploration and potential integration into therapeutic strategies.

### 3.2. Belzutifan Plus CDK4/6 Inhibitors

The combination of belzutifan with cyclin-dependent kinase (CDK) 4/6 inhibitors, such as palbociclib and abemaciclib, is emerging as a potentially effective therapeutic approach for metastatic ccRCC. This strategy leverages biological interactions due to shared pathways between these agents. VHL gene loss-of-function mutations, frequently observed in ccRCC, lead to HIF-2α activation, which subsequently increases cyclin D1 expression. Cyclin D1 then partners with CDK4/6 to stimulate tumor cell proliferation. Preclinical studies have demonstrated that pairing a HIF-2α inhibitor like PT2399 with a CDK4/6 inhibitor produces synergistic anti-tumor effects in ccRCC cell lines and VHL-deficient xenograft models provide a robust rationale for the ongoing investigation of belzutifan and CDK4/6 inhibitors in the treatment of metastatic RCC.

By targeting both HIF-2α and CDK4/6, the therapy aims to disrupt cancer progression on multiple fronts.

In the randomized phase I/II trial LITESPARK-024 trial (NCT05468697), belzutifan combined with palbociclib is compared to belzutifan alone in patients with advanced ccRCC who have progressed after standard treatments, including immunotherapy and VEGF inhibitors. Preliminary findings from the phase I/II trial indicate encouraging response rates, though comprehensive data on efficacy and survival outcomes are pending. Key endpoints include ORR, OS, and PFS, with a particular focus on the combination’s safety profile given the dual mechanism of action.

The study has two main parts: phase I (dose escalation) aims to establish the safety of the belzutifan–palbociclib combination and determine the recommended dose of palbociclib for subsequent testing and phase 2 (randomized cohort) evaluates the efficacy and safety of the recommended dose combination versus belzutifan monotherapy. The primary outcome for efficacy is based on PFS, with secondary measures including ORR and safety profiles.

Additionally, the ongoing phase Ib trial (NCT04627064) explores belzutifan with abemaciclib in a similar patient population. This study seeks to determine optimal dosing and evaluate ORR as a primary outcome, alongside secondary measures like safety and duration of response. Early observations suggest a feasible therapeutic window, but further analysis is needed to establish potential advantages over current standards and validate the hypothesized synergy.

The trial enrolled a limited cohort of 11 patients, and despite the high level of pre-treatment, abemaciclib monotherapy did not show significant anti-tumor activity. Treatment-related AEs were common, with most participants experiencing mild to moderate side effects like increased creatinine levels and gastrointestinal disturbances. No severe, life-threatening toxicity signals were observed, but patient-reported outcomes suggested that the treatment was burdensome.

These studies reflect an evolving treatment landscape in metastatic ccRCC, where combined approaches targeting both hypoxic and cell-cycle dysregulation pathways could offer novel, effective options for patients with limited responses to traditional therapies.

### 3.3. Belzutifan Plus TKI

The combination of belzutifan with TKIs such as cabozantinib (against VEGF receptor (VEGFR), c-MET, AXL, and RET) and lenvatinib (VEGFR1-3, c-Kit, fibroblast growth factor receptors (FGFRs)1-4, platelet-derived growth factor receptors (PDGRs)-α, and RET) has shown promising efficacy in clinical trials for patients with advanced ccRCC.

The phase II LITESPARK-003 trial demonstrated considerable anti-tumor activity for the belzutifan and cabozantinib combination. In treatment-naïve patients from cohort 1, with a median follow-up of 28.6 months, an ORR of 70% was achieved, along with a median PFS of 30.3 months. Among previously treated patients in cohort 2, with a median follow-up of 31.5 months, the ORR reached 31%, and the median PFS was 13.8 months. The duration of response averaged 28.6 months in treatment-naïve patients and 31.5 months in previously treated individuals. While the median OS was not reached in treatment-naïve patients, it was 26.7 months in those who had undergone prior treatment. The safety profile was manageable, though notable AEs included hypertension and fatigue, with 63% experiencing grade 3–5 treatment-related effects in cohort 2, and some patients required dose adjustments due to toxicity.

In the KEYMAKER-U03B phase I/II study (NCT04626518), early results showed an ORR of 50% in patients with metastatic RCC who had previously received both immunotherapy and VEGF-TKI treatment. With a median follow-up period of almost 6 months, the median PFS was reported at 11.2 months, with a 6-month PFS rate of 55%. The most common AEs included anemia, fatigue, and hypertension, each occurring in 43% of patients.

NCT04586231 is an ongoing phase III randomized, open-label study comparing the efficacy of belzutifan combined with lenvatinib against cabozantinib in patients with advanced ccRCC who have progressed after prior anti-PD-1/L1 therapy. This trial aims to demonstrate that combination therapy improves PFS and OS compared to cabozantinib alone. A total of 708 participants will be enrolled and randomly assigned to one of the two treatment groups. The study’s design emphasizes evaluating the clinical benefits of belzutifan and lenvatinib, particularly in a population with limited treatment options following anti-PD-1/L1 therapy.

These studies represent a significant step forward in exploring the potential of combining novel agents like belzutifan with established TKIs to enhance treatment outcomes in advanced RCC, a cancer type that often presents significant therapeutic challenges. Both trials are part of ongoing efforts to develop effective second-line treatments for RCC patients, particularly those who have previously undergone immunotherapy.

### 3.4. Belzutifan Plus Immune Checkpoint Inhibitors

Under hypoxic conditions, not only is HIF signaling activated, but PD-L1 expression is also elevated, indicating cross-talk between HIF pathways and immune responses in tumors. Elevated HIF-2α levels have been linked to reduced infiltration of CD8+ T lymphocytes in tumors, alongside the production of stem cell factors (SCFs). SCFs subsequently promote the release of IL-10 and TGF-β, which together help establish an immunosuppressive tumor microenvironment. This suggests that combining HIF-2α inhibitors with ICIs may yield synergistic effects that could enhance anti-tumor immunity [29,30]. The combination of belzutifan with ICIs is under rigorous investigation across multiple clinical trials, aiming to exploit the unique synergistic potential of HIF-2α inhibition with immune modulation.

An ongoing phase III study is evaluating the efficacy of pembrolizumab plus belzutifan and lenvatinib against the standard pembrolizumab–lenvatinib combination in advanced ccRCC (NCT04736706). This trial will assign patients with previously untreated metastatic ccRCC to one of three regimens: a combination of belzutifan, lenvatinib, and pembrolizumab; a regimen of belzutifan, lenvatinib, and quavonlimab (an anti-CTLA-4); or lenvatinib with pembrolizumab alone [31]. The primary endpoints are PFS and OS. Preliminary data indicate that the triplet therapy has shown encouraging signals of improved PFS, suggesting that the inhibition of HIF-2α could augment the immunomodulatory and antiangiogenic effects of pembrolizumab and lenvatinib. The safety profile of the triplet regimen includes notable AEs such as hypertension and anemia, reflective of the individual drug toxicity profiles but managed through established protocols.

Trials are currently underway to assess the triple combination of belzutifan, lenvatinib, and pembrolizumab in other studies (NCT05899049 and NCT05030506). Additionally, the ongoing LITESPARK-022 trial (NCT05239728), a phase III study, is investigating the combination of pembrolizumab with either belzutifan or a placebo as adjuvant therapy for patients with ccRCC who are at high risk for recurrence following nephrectomy or metastasectomy. This trial’s primary endpoint is disease-free survival (DFS), with secondary endpoints that include OS and safety metrics.

Meanwhile, another phase Ib/II study (NCT04626479) is exploring the use of a co-formulation of vibostolimab and pembrolizumab, alongside belzutifan, as a first-line treatment for metastatic RCC.

These trials collectively underscore the strategic rationale of combining HIF-2α inhibition with immune checkpoint blockade. By simultaneously targeting tumor hypoxia and leveraging immune activation, researchers hope to achieve more durable and potent anti-cancer responses in patients with limited treatment options. The ongoing analyses will clarify the long-term benefits and any emerging safety concerns associated with these novel regimens.

Table 1 summarizes all ongoing trials involving anti-HIF-2α in RCC treatments.

### 3.5. Other Combinations

Other new molecules are evaluating the combination with HIF-2 α inhibitors. For instance, the phase Ib open-label (NCT05121948) multicenter study is evaluating the safety, tolerability, and preliminary efficacy of HC-7366 both as a monotherapy and in combination with belzutifan. HC-7366, a first-in-class, first-in-human modulator of general control nonderepressible 2 (GCN2) with the capacity to activate GCN2, demonstrates potential anti-tumor activity.

Preclinical studies have shown that HC-7366 exhibits notable efficacy across multiple solid tumor types as well as in acute myeloid leukemia (AML) models.

This study includes three study arms: an HC-7366 monotherapy cohort, a combination dose-escalation phase, and a combination dose-expansion phase (NCT05121948). Aiming to enroll approximately 80 patients, the study will allocate up to 20 patients to the monotherapy cohort, 30 to the combination dose-escalation phase, and another 30 to the dose-expansion phase. The primary objective of the trial is to identify the maximum tolerated dose (MTD) of HC-7366 in combination with belzutifan for patients with locally advanced or metastatic RCC with predominantly clear cell histology, regardless of VHL gene mutation status.

## 4. Resistance Mechanisms of HIF-2α-Targeted Therapies

To date, several potential mechanisms have been proposed to explain resistance to HIF inhibition. Two studies demonstrated that higher expression of HIF1A encoding for HIF-1α and lower HIF-2α levels are associated with resistance to PT2399 (a derivative of belzutifan) in RCC models [32,33]. In another study performed with xenograft RCC tumors, the G323E mutation in endothelial PAS domain-containing protein 1 (EPAS1) seems to be related to the resistance to PT2399 treatment [33]. EPAS1 G323E mutation and subsequently HIF-2 inactivation always seem to be related to the progression of a first-generation HIF-2α inhibitor (PT2385) in patients treated in a phase I trial; this mutation may occur at baseline and after treatment initiation. Interestingly, the same study showed a role of TP53 in a resistance mechanism to HIF-2α-targeted therapies [34]. However, another study did not confirm the role of TP53 in resistance to HIF-2α inhibitors [35]. Finally, the HIF-1/ARNT complex has been showed as a possible resistance mechanism with HIF-2α inhibitors [33,36]. Unfortunately, to date, the precise mechanism of resistance to HIF-2α inhibitors has not been discovered; for this reason, to overcome belzutifan resistance, studies with novel HIF-2α-targeting strategies or combinatorial approaches are awaited.

## 5. New Anti HIF-2 Inhibitors

The development of belzutifan has inspired a new generation of HIF-2α inhibitors in RCC research, with a variety of novel agents advancing through preclinical and clinical testing stages. Some of these agents, distinct from belzutifan in their mechanisms and targets, are showing early promises in both safety and efficacy.

DFF332, a HIF-2α inhibitor operates by disrupting the HIF pathway, a known driver of tumor growth and survival in hypoxic environments, such as those often found in RCC.

The clinical trial NCT04895748 is a phase I study evaluating the safety and efficacy of DFF332, either as a single agent or in combination with other therapies like spartalizumab (anti-PD-1) and taminadenant, an adenosine A2A receptor antagonist. This trial primarily targets patients with advanced or relapsed ccRCC and other malignancies that feature mutations stabilizing the HIF-2α. The trial is designed to assess the compound’s safety, with endpoints that include the determination of dose-limiting toxicities and optimal dosage, as well as preliminary efficacy markers such as ORR and PFS.

Another promising agent is ARO-HIF2, a targeted RNA interference therapy. ARO-HIF2 targets HIF-2α through small interfering RNA (siRNA) technology, which has shown a reduction in HIF-2α levels and tumor size in preclinical RCC models. In a phase I trial for previously treated mRCC patients (NCT04169711), ARO-HIF2 demonstrated a DCR of 30%, with fatigue as a common side effect, and a few serious AEs, including hypoxia and myocarditis. The study provided early proof of concept for RNA-based therapies, indicating potential for future development.

A novel oral HIF-2α inhibitor NKT-2152 is being studied as a single agent and in combination therapies. At the ESMO 2024 Congress, the results of the phase I/II trial (NCT05119335) investigating the efficacy and safety of NKT2152, in patients with advanced ccRCC who had received at least one prior line of therapy, have been reported. A total of 113 patients were enrolled, with 73 in the dose-escalation and pharmacokinetic phase and 40 in the expansion phase. The overall ORR was 20%, with a median PFS of 7.5 months. Among patients with no prior mTOR inhibitor treatments, the ORR was 40%, and PFS reached 9.4 months. The maximum tolerated dose was not reached, and the drug exhibited a linear and time-independent pharmacokinetic profile, with notable suppression of erythropoietin. Grade 3 or higher adverse events included hypoxia (16%), anemia (15%), and fatigue (5%).

The phase II clinical study is designed to assess the safety and efficacy of the HIF-2α inhibitor NKT2152 in combination therapies for patients with advanced or metastatic ccRCC who have previously undergone treatment. In the study’s lead-in phase, researchers aim to evaluate the safety, therapeutic effects, and pharmacokinetics (PK) of NKT2152 when administered with palbociclib and with both palbociclib and sasanlimab, ultimately establishing a recommended dose for expansion (RDE). In the expansion phase, the study will continue to monitor safety, efficacy, and PK at the selected RDE, focusing on determining the recommended phase II dose (RP2D) for each combination in patients with advanced or metastatic ccRCC who have received prior therapies.

BPI-452080 is a selective HIF-2α inhibitor that prevents the dimerization of HIF-2α with HIF-1β, leading to lower transcription of hypoxia-responsive genes like GLUT1, CCND1, and CXCR4, and reducing VEGFA secretion in RCC preclinical models. Oral administration of BPI-452080 in xenograft models has shown a dose-dependent decrease in tumor size. As of early 2023, a phase I clinical trial (NCT05843305) is underway in China to evaluate BPI-452080’s safety and effectiveness in patients with mRCC, including those with VHL-associated mRCC, as well as in other advanced solid tumors.

Similarly, AB521 is a novel inhibitor targeting HIF-2α that blocks the interaction between HIF-2α and HIF-1β, thereby interfering with the expression of genes regulated by HIF-2α. This inhibitor has demonstrated dose-dependent tumor reduction in preclinical RCC xenograft studies. Additionally, preclinical models suggest that combining AB521 with cabozantinib may have synergistic effects, enhancing anti-tumor activity compared to the efficacy of each drug alone. In a phase I study focused on pharmacokinetics and pharmacodynamics, AB521 reduced circulating erythropoietin levels proportionally to the dose. This drug is currently under investigation in a phase I trial (NCT05536141), aimed at assessing safety and efficacy in patients with metastatic RCC who have already undergone anti-PD-1 or TKI therapies, as well as in other advanced solid tumor cases.

Lastly, KD061 represents an emerging approach to HIF inhibition through the induction of ferroptosis, a cell death pathway distinct from traditional apoptosis. KD061, shown to decrease tumor growth in preclinical RCC models, targets iron–sulfur cluster assembly proteins, suggesting that combining HIF inhibition with ferroptosis may improve outcomes in HIF-resistant cancers, with potential for future clinical exploration.

These studies collectively illustrate a diverse landscape of HIF-2α inhibitors in RCC, exploring innovative mechanisms to improve therapeutic responses and overcome resistance seen in monotherapies.

## 6. Conclusions and Future Directions

Recently, the therapeutic scenario of mRCC has been revolutionized by the possibility of combining antiangiogenic therapy with immunotherapy. Based on these premises, targeting HIF-2α seems very promising because HIF-2α inhibitors work with a novel mechanism of action that is potentially able to overcome resistance to immunotherapy and/or antiangiogenetic drugs. Another attractive way is to combine HIF-2α inhibitors with active or experimental drugs; finally, it is very intriguing to explore the use of HIF-2α inhibitors in first-line or adjuvant settings. For all these reasons, results from clinical trials and the recognition of the potential mechanisms of resistance to HIF-2α inhibitors are urgently awaited. To date, there is a need to determine the optimal sequencing of all available drugs for mRCC; in this context, HIF-2α inhibitors alone or in combination may have the greatest clinical benefit in first-line or subsequent settings, such as an adjuvant setting, with the possibility of a rechallenge, or they should be continued beyond progression such as in other target therapies [37]. However, belzutifan is currently the only anti-HIF drug that has received FDA approval, marking a significant milestone in the field, while other HIF-targeted drugs remain under clinical investigation, with varying stages of progress. For instance, several are in phase 2 or phase 3 trials, and early data suggest they could hold promise within the next 5–10 years, depending on regulatory approval and the outcome of ongoing studies. It is therefore reasonable to anticipate that broader adoption of HIF-targeted therapies may require more extended timelines for development and validation.

Another field of interest should be focused on the investigation of newer-generation HIF-2α inhibitors able to overcome the EPAS1 G323E resistance mutation that seems to be related to the progression of first-generation HIF-2α inhibitors.

However, in addition to their therapeutic potential, HIF-targeted treatment strategies exhibit pleiotropic effects that warrant careful consideration, particularly given their broad biological implications. HIF inhibitors influence multiple downstream pathways, including angiogenesis, metabolism, and immune modulation, which can lead to off-target effects or unintended consequences in non-malignant tissues. These effects may pose risks such as impaired wound healing, cardiovascular complications, or metabolic disturbances, depending on the patient’s underlying conditions.

Moreover, certain populations may face heightened risks, necessitating attention to contraindications. For example, patients with pre-existing vascular or metabolic disorders, or those requiring robust tissue repair mechanisms, might not tolerate these treatments well. Careful patient selection and risk stratification are therefore critical to optimizing the benefit–risk profile of HIF-targeted therapies.

Future research should aim to elucidate the full spectrum of pleiotropic effects, refine biomarkers for predicting treatment responses, and develop mitigation strategies to minimize risks while maintaining therapeutic efficacy.

Unfortunately, to date, no predictive biomarkers are available for the selection of therapies in RCC; for example: VHL, VEGF, PD-L1, and other possible biomarkers failed to clearly show a possible predictive role in RCC and, to date, only clinical characteristics are used to guide the selection of patients to treat with an immune combo or TKI alone [38].

For all these reasons, in the near future, the efforts of preclinical and clinical research should be directed towards the selection of a subgroup of patients that may or may not respond to standard therapy but also HIF-2α inhibitors.

## Figures and Tables

**Table 1:** Clinical trials involving anti-HIF-2α in renal cell carcinoma treatment.

Study (number)	Phase	Patients	Enrollment Estimated	Experimental Drug	Start Date	Status
NCT04846920 (LITESPARK-018)	Phase I	Advanced ccRCC	52	Belzutifan	14 June 2021	Active not recruiting
NCT04489771 (LITESPARK-013)	Phase II	Advanced RCC	154	Belzutifan	13 September 2020	Active not recruiting
NCT06234605	Phase Ib	Advanced RCC	80	HC-7366 plus Belzutifan	29 April 2024	Recruiting
NCT04195750 (LITESPARK-005)	Phase 3	Advanced RCC	755	Belzutifan versus Everolimus	27 February 2020	Active not recruiting
NCT05468697 (LITESPARK-024)	Phase I/II	Advanced RCC	210	Belzutifan plus Palbociclib versus Belzutifan Monotherapy	10 August 2022	Recruiting
NCT05239728 (LITESPARK-022)	Phase III	ccRCC post-Nephrectomy	1800	Belzutifan plus Pembrolizumab versus Placebo plus Pembrolizumab	15 March 2022	Active not recruiting
NCT03634540 (LITESPARK-003)	Phase II	ccRCC	118	Belzutifan plus Cabozantinib	27 September 2018	Active not recruiting
NCT04586231	Phase III	Advanced RCC	708	Belzutifan plus Lenvatinib versus Cabozantinib	25 February 2021	Active not recruiting
NCT05030506 (LITESPARK-010)	Phase I	Advanced RCC	45	Belzutifan as Monotherapy plus Lenvatinib with or without Pembrolizumab	13 October 2021	Active not recruiting
NCT05899049	Phase III	Advanced ccRCC-China Extension Study	249	Pembrolizumab plus Belzutifan and Lenvatinib, or Pembrolizumab/Quavonlimab plus Lenvatinib, versus Pembrolizumab and Lenvatinib	27 July 2022	Active not recruiting
NCT04736706	Phase III	Advanced ccRCC	1653	Pembrolizumab plus Belzutifan and Lenvatinib, or Pembrolizumab/Quavonlimab plus Lenvatinib, versus Pembrolizumab and Lenvatinib	14 April 2021	Active not recruiting
NCT02974738 (LITESPARK-001)	Phase I	Advanced Solid Tumors	120	Belzutifan Tablets	7 December 2016	Active not recruiting
NCT03401788 (LITESPARK-004)	Phase II	Von Hippel–Lindau (VHL) Disease-Associated RCC (MK-6482-004)	50	Belzutifan (PT2977, MK-6482)	2 May 2018	Active not recruiting
NCT04626518 (KEYMAKER-U03B) Substudy 03B	Phase I/II	Second-Line Plus (2L+) RCC	370	Immune and Targeted Combination Therapies	17 December 2020	Active not recruiting
NCT04626479 (KEYMAKER-U03B) Substudy 03A	Phase I/II	First-Line (1L) RCC	400	Immune and Targeted Combination Therapies	16 December 2020	Active not recruiting
NCT04627064	Phase Ib	ccRCC Sarcomatoid RCC	40	Abemaciclib Monotherapy or in Combination with MK-6482	31 December 2020	Active not recruiting
NCT02293980	Phase I	Advanced ccRCC	110	PT2385 Tablets	25 November 2014	Active not recruiting

Clear cell renal cell carcinoma: ccRCC.

## Data Availability

No new data were created or analyzed in this study. Data sharing is not applicable to this article.

## Glossary Abbreviation

AEs	adverse events
ATP	adenosine triphosphate
AUC	area under the plasma concentration–time curve
ccRCC	clear cell RCC
CDK	cyclin-dependent kinase
CTLA-4	cytotoxic T-lymphocyte antigen 4
DCR	disease control rate
DFS	disease-free survival
EORTC QLQ-C30	European Organisation for Research and Treatment of Cancer Core Quality of Life questionnaire
EPAS1	endothelial PAS domain-containing protein 1
EPO	erythropoietin
FGFRs	fibroblast growth factor receptors
FKSI-DRS	The Functional Assessment of Cancer Therapy–Kidney Symptom Index—Disease-Related Symptoms
GCN2	general control nonderepressible 2
GLUT1	glucose transporter 1
HIF	hypoxia-inducible factor
HREs	hypoxia-responsive elements
HRQoL	health-related quality of life
ICI	immune checkpoint inhibitor
mRCC	metastatic RCC
MTD	maximum tolerated dose
ORR	overall response rate
OS	overall survival
PD-1	programmed death 1
PD-L1	programmed death-ligand 1
PDGFRs	platelet-derived growth factor receptors
PFS	progression-free survival
PHD	prolyl hydroxylase domain
PK	pharmacokinetics
PPGLs	pheochromocytomas/paragangliomas
RCC	renal cell carcinoma
RDE	recommended dose for expansion
RP2D	recommended phase II dose
SCF	stem cell factor
siRNA	small interfering RNA
TKIs	tyrosine kinase inhibitors
VEGF	vascular endothelial growth factor
VEGFR	vascular endothelial growth factor receptor
VHL	von Hippel–Lindau
